# Nursing Students’ Perceptions on Healthcare-Associated Infection Control and Prevention Teaching and Learning Experience in Portugal

**DOI:** 10.3390/jpm12020180

**Published:** 2022-01-28

**Authors:** Pedro Parreira, Paulo Santos-Costa, João Pardal, Teresa Neves, Rafael A. Bernardes, Beatriz Serambeque, Liliana B. Sousa, João Graveto, Marja Silén-Lipponen, Ulla Korhonen, Leena Koponen, Mikko Myllymäki, Amaia Yurrebaso Macho, Alexander L. Ward Mayens, Eva Maria Picado Valverde, Raquel Guzmán Ordaz, Juan Antonio Juanes Méndez, Jose Luis Pérez Iglesias, José Antonio Mirón Canelo, Aleksandra Jankowiak-Bernaciak, Amelia Patrzała, Grażyna Bączyk, Anna Basa, Alcinda Maria do Sacramento Costa Reis, Joaquim Augusto Simões, Ana Luísa Torres, Maria do Rosário Pinto, Anabela Salgueiro-Oliveira

**Affiliations:** 1 The Health Sciences Research Unit: Nursing (UICISA: E), Nursing School of Coimbra (ESEnfC), 3004-011 Coimbra, Portugal; parreira@esenfc.pt (P.P.); jgrpardal@esenfc.pt (J.P.); teresa_neves@esenfc.pt (T.N.); rafaelalvesbernardes@esenfc.pt (R.A.B.); beatrizprazserambeque@esenfc.pt (B.S.); baptliliana@esenfc.pt (L.B.S.); jgraveto@esenfc.pt (J.G.); anabela@esenfc.pt (A.S.-O.); 2 Coimbra Institute for Clinical and Biomedical Research (iCBR) Area of Environment Genetics and Oncobiology (CIMAGO), University of Coimbra, 3000-548 Coimbra, Portugal; 3 Institute of Biophysics, Faculty of Medicine, University of Coimbra, 3000-548 Coimbra, Portugal; 4 Center for Innovative Biomedicine and Biotechnology (CIBB), University of Coimbra, 3000-548 Coimbra, Portugal; 5 Savonia University of Applied Sciences, Health and Social Care, 70201 Kuopio, Finland; marja.silen-lipponen@savonia.fi (M.S.-L.); ulla.korhonen@savonia.fi (U.K.); leena.koponen@savonia.fi (L.K.); mikko.myllymaki@savonia.fi (M.M.); 6 Salamanca University, 37008 Salamanca, Spain; amaiay@usal.es (A.Y.M.); alexander.ward456@gmail.com (A.L.W.M.); evapicado@usal.es (E.M.P.V.); r.guzman@usal.es (R.G.O.); jajm@usal.es (J.A.J.M.); jpi@usal.es (J.L.P.I.); miroxx@usal.es (J.A.M.C.); 7 Hipolit Cegielski State University of Applied Sciences in Gniezno, 62-200 Gniezno, Poland; a.jankowiak-bernaciak@pwsz-gniezno.edu.pl (A.J.-B.); a.patrzala@pwsz-gniezno.edu.pl (A.P.); g.baczyk@pwsz-gniezno.edu.pl (G.B.); a.basa@pwsz-gniezno.edu.pl (A.B.); 8 Department of Practice Nursing, Poznan University of Medical Sciences, 61-701 Poznan, Poland; 9 Escola Superior de Saúde, IP Santarem, 2040-413 Santarém, Portugal; alcinda.reis@essaude.ipsantarem.pt (A.M.d.S.C.R.); joaquim.simoes@essaude.ipsantarem.pt (J.A.S.); ana.torres@ese.ipsantarem.pt (A.L.T.); mrosario.pinto@esel.pt (M.d.R.P.)

**Keywords:** nursing students, healthcare-associated infections, education

## Abstract

Healthcare-associated infections (HAI) are one of the major concerns worldwide, posing significant challenges to healthcare professionals’ education and training. This study intended to measure nursing students’ perceptions regarding their learning experiences on HAI prevention and control. In the first phase of the study, a cross-sectional and descriptive study with a convenience sample composed of undergraduate nursing students from Portugal, Spain, Poland, and Finland was conducted to develop the InovSafeCare questionnaire. In the second phase, we applied the InovSafeCare scale in a sample of nursing students from two Portuguese higher education institutions to explore which factors impact nursing students’ adherence to HAI prevention and control measures in clinical settings. In phase one, the InovSafeCare questionnaire was applied to 1326 students internationally, with the instrument presenting adequate psychometric qualities with reliability results in 14 dimensions. During phase two, the findings supported that Portuguese nursing students’ adherence to HAI prevention and control measures is influenced not only by the curricular offerings and resources available in academic settings, but also by the standards conveyed by nursing tutors during clinical placements. Our findings support the need for a dedicated curricular focus on HAI prevention and control learning, not only through specific classroom modules, innovative resources, and pedagogical approaches, but also through a complementary and coordinated liaison between teachers and tutors in academic and clinical settings.

## 1. Introduction

Healthcare-associated infections (HAI) is the contemporary term used to refer to infections that occur as the result of healthcare interventions [1] and were not present or incubating at the time of care delivery but could be established 48 h or more after. HAI represent one of the most frequent and serious complications in healthcare settings, from primary care to hospitals [2,3]. 

Presently, HAI are one of the biggest challenges for healthcare professionals and managers, directly influencing patients’ morbidity and mortality rates, delaying discharges, and increasing care costs with proper diagnosis and treatment [4]. Among the factors that can influence the occurrence of this infection is the susceptibility of the host, the environment, and the influence of care practices by healthcare professionals [5,6]. The current prevalence rate of HAI in developed countries is around 7% and in developing countries it is around 10% [7,8]. The European Center for Disease Prevention and Control estimates that up to 4.6 million people acquire a HAI each year in acute care settings, which represents the most deadly and costly adverse event in public hospitals across Europe [9]. Despite such data, most HAI are avoidable through the creation of local infection control teams, use of evidence-based guidelines, infection surveillance and feedback, as well as thorough maintenance of environmental hygiene [9]. Whatever strategies are used to reduce the burden of HAI, they will only be effective if all people involved in patient care and treatment, including healthcare students, have a thorough grasp of the current prevention and control measures [10]. 

To address this challenge, the World Health Organization emphasized the importance of formally including HAI prevention and control modules in healthcare students’ undergraduate courses, thereby training them to be active agents of change in clinical practice. Amongst them, nursing students’ education and training in this field are essential to ensure the quality and safety of the care provided [8,11]. Nursing students play a critical role in the spread of pathogens and the prevention of infectious diseases as members of the healthcare team, as they treat and care for a wide range of patients throughout their clinical placement [11].

In Europe, nursing is a practice-oriented higher education course. Approximately half of the training hours must be developed in a clinical setting, oriented towards direct contact with people in need of nursing care [12,13]. During their clinical placements, nursing students are expected to demonstrate their professional values, knowledge, and competencies in areas such as decision-making, interpersonal relationships, communication, leadership, management, and creativity [13,14]. Thus, the existence of dedicated courses on HAI prevention and control in undergraduate nursing courses is essential to guarantee their awareness and preparedness in the future [10].

However, the nursing curriculum is not standardized between European nursing HEIs [14], and there are no clear recommendations in the European Higher Education Area concerning the importance of having dedicated courses on HAI prevention and control (e.g., a dedicated number of hours or mandatory curricular content). This can lead to inconsistencies or omissions in how nursing students learn about this subject in different European HEIs.

Thus, this study aimed to evaluate and explore the nursing student’s perceptions regarding their teaching and learning experience in the field of HAI prevention and control in two Portuguese Higher Education Institutions (HEIs).

## 2. Materials and Methods

A quantitative, cross-sectional study was conducted in two phases: (i) questionnaire development and validation; and (ii) path analysis with Portuguese nursing students.

### 2.1. Phase 1—Questionnaire Development and Validation

The validation process of the InovSafeCare scale followed a mixed approach of qualitative and quantitative methods, which was subjected to previous publication [15]. 

First, after a comprehensive literature review, a panel of researchers identified the most mentioned HAI-related dimensions in this thematic field, creating a list of items in English (α version). To ensure the instrument was scientifically sound and applicable across different cultural settings, the α version was reviewed by a panel of international subject matter experts (SMEs) in the fields of nursing and HAI prevention and control. Through several rounds of virtual meetings, the panel members provided their feedback on each item for grammar, redundancy, latent factor correspondence, context correction, and relevance. 

After an agreed version was obtained, to ensure that the items were equally applicable and understood by nursing students from different countries, the questionnaire was submitted to a cultural adaptation procedure. The β version (in English) was then translated and culturally adapted to the nursing student population from Portugal, Spain, Poland, and Finland according to Beaton and colleagues’ recommendations for self-reported measures [16]. 

The validation of the instrument was conducted using a multicentric approach and a nonprobability convenience sampling technique was used. The questionnaire was applied to undergraduate nursing students from Portugal (Nursing School of Coimbra and Polytechnic School of Health of Santarém, Portugal), Poland (Hipolit Cegielski Higher School in Gniezno), Spain (University of Salamanca), and Finland (Savonia University of Applied Sciences). The psychometric properties of the scale were assessed through a partial least square structural equation modeling (PLS-SEM) method. Data was collected between October 2019 and March 2020. 

### 2.2. Phase 2—Path Analysis with Portuguese Nursing Students

A multiple regression model (path analysis) was used to analyze data and measure the direct effects on deviations in good practices related HAI prevention. The path analysis is an extension of the multivariate multiple regression analysis, which was used to estimate the effects of different factors on the deviation in good practices related to healthcare-associated infection among undergraduate nursing students. The significance of the regression coefficients was determined after the estimation of the parameters through the maximum likelihood estimation method. The significance of the effects (direct, indirect, and total), to confirm the mediation effect, was determined by the bootstrap resampling method (2000 samples and 90% bias-corrected CI) using AMOS software (version 22, An IBM Company, Chicago, IL, USA). 

The model fit was estimated through the maximum-likelihood estimation method using AMOS software (version 22, An IBM Company, Chicago, IL, USA). Statistically significant effects were assumed for *p* ≤ 0.05. We used different goodness-of-fit indices and were considered acceptable values of χ2/df < 5, CFI and GFI > 0.90, and RMSEA < 0.08. The significance of the direct, indirect, and total effects was measured showing the significance of the regression coefficients. This path analysis focused on the variables that could influence the students’ practices in healthcare-associated infection. In the path analysis model, the trajectories which were not statistically significant were excluded.

A descriptive analysis (measures of central tendency, dispersion, and frequency) was also performed using the Statistical Package for the Social Sciences (version 22.0, SPSS An IBM Company, Chicago, IL, USA).

### 2.3. Ethical Considerations 

This study received a positive review from the Ethical Committee of The Health Sciences Research Unit: Nursing (UICISA: E) of the Nursing School of Coimbra (reference 635/11-2019). The participants’ anonymity and individual data confidentiality were preserved. Following the ethical principles of the Declaration of Helsinki, students were formally introduced to the study goals by one element of the research team. All students who agreed to participate signed an informed consent form. Anonymity and confidentiality were maintained through the study. All data collection instruments used during this study were alphanumerically encoded to preserve the students’ identity.

## 3. Results

### 3.1. Phase 1—Questionnaire Development and Validation

The international convenience sample was composed of 1326 nursing students from Portugal, Spain, Poland, and Finland. The InovSafeCare questionnaire showed good validity and reliability of the dimensions in different European countries with adequate composite reliability (CR) and variance extracted (AVE). The psychometric qualities of the questionnaire supported the item pool related to the educational environment, healthcare setting environment, attitudes, beliefs, and performance of the nursing students regarding HAI prevention and control. The resulting scale named InovSafeCare questionnaire led to 93 items representing the following 14 dimensions (Table 1). 

The Portuguese sample has a similar distribution with less discrimination, denoting, however, a lower percentage of answers in the minimum of the Likert scale in some of the items, as presented in Table 2.

The questions in QVII, QXI, and QXIII group’s present mean values below the midpoint of the scale, which was to be expected given that they report to nursing students’ distraction, personal objection to HAI prevention and control measures, and deviation in good practices in this field. The results show adequate reliability results in the 14 dimensions, as presented in the Table 3.

### 3.2. Phase 2—Path Analysis with Portuguese Nursing Students

The assumptions of normality and multicollinearity were confirmed, and the values indicated no severe violation of the normality assumption. The variance inflation factor values were below five. The squared Mahalanobis distance values suggested 18 outliers that were removed.

In the variables under analysis, adherence to a normal distribution (skewness (Sk) and kurtosis (Ku) values indicated no severe violation of the normality assumption (|Sk| < 3 and |Ku| < 7). None of the variables had multicollinearity problems or sufficiently strong correlations among the exogenous variables. 

The model was adjusted to self-reported HAI practices according to the Higher Education Institution (theoretical content, approach to teaching, and HAI practice-related approach/resources) and Health Institution (HAI prevention practices and culture), especially considering that a set of mediating variables explains 22% of the variability in the student’s self-reported HAI practices. 

**Figure 1:**
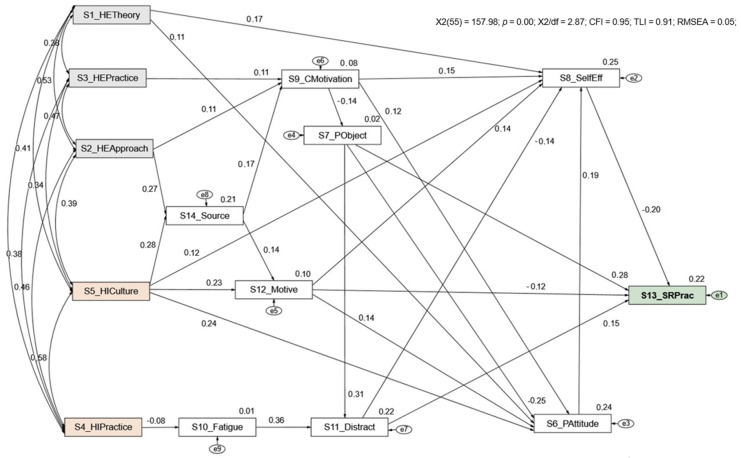
Multifactorial path analysis model of the standardized effect of factors determining the students self-reported HAI practices. Healthcare-associated infections (HAI).

Figure 1 shows the model with the standardized regression coefficients and the R2 of the student’s self-reported HAI practices. The model has a good goodness-of-fit (χ2/df(55) = 2.87; CFI = 0.95, TLI = 0.91, RMSEA = 0.05).

The results show that all model trajectories are statistically significant (*p* ≤ 0.05), such as the Higher Education Institution HAI Theoretical Content “HETheory”, Higher Education Institution Approach to teaching HAI “HEApproach”, and Higher Education Institution HAI practice-related approach/resources “HEPractice” (total effect: βHETheory = −0.04; βHEApproach = −0.02; βHEPractice = −0.01); moreover, HIPractice and HICulture (total effect: βHAI HIPractice practices = −0.01; βHICulture = −0.08) have a significant indirect effect on students’ self-reported HAI practices (SRPract). Sources of knowledge acquired outside the institution “Sources” and factors such as “Fatigue”, “Motivation”, and personal HAI attitudes “Patitude” mediated this relationship and have an indirect effect on the students’ self-reported HAI practices “SRPract”. The dimension’s “Distraction”, personal objection to HAI prevention and control measures “PObject”, motives to comply with HAI control and prevention measures “IMotive”, and self-efficacy on HAI prevention and control “SelfEff”, in addition to mediating, have a significant direct effect on the students’ self-reported HAI practices “SRPract”. 

The Higher Education Institution HAI theoretical content “HETheory” directly influences the personal HAI attitudes “PAttitude” (β = 0.11) and self-efficacy on HAI prevention and control “SelfEff” (β = 0.17). It is also noted that the approach to teaching HAI “HEApproach” and HAI practice-related approach/resources of Higher Education Institutions “HEPractice” foster Student’s motivation “CMotivation”. Based on the bootstrap resampling method, all effects are significant (*p* ≤ 0.05). 

## 4. Discussion

Nursing students’ learning experiences about HAI prevention and control is still an understudied area, with several international authors identifying substandard knowledge levels and attitudes towards this field [17,18,19]. Such results are disheartening since nurses compose the largest professional group in healthcare, with a significant role in preventing HAI. In this study, we identified several areas that may explain how nursing students in Portugal perceive their learning and teaching experiences concerning HAI prevention and control. Our findings show that nursing students’ adherence to HAI prevention and control involves a myriad of factors that can be attributed to current pedagogical practices in both academic and clinical settings.

Concerning their learning and teaching experiences in academic settings, nursing students’ motivation and positive attitudes towards HAI prevention and control is highly associated with the existence of specific theoretical and practical classes in this field. Interestingly, the more robust the pedagogical offering in this area, the greater their motivation was for a future career in nursing (β = 0.11). Students’ motivation seems to be positively influenced by the existence of sufficient theoretical–practical lessons on this topic, along with an adequate teacher–student ratio and adequate number of resources in laboratory sessions. Likewise, implementing simulation-based scenarios in HAI prevention and control seems to be another well-received pedagogical strategy that contributes to students’ self-efficacy in this field, which converges with results from previous studies [20,21,22]. 

Although such results converge with recent literature on the topic, it emphasizes the demand felt by Portuguese nursing students to have specific curricular units on HAI prevention and control. This can be a challenge for several nursing HEIs in Portugal since this topic is often lectured on in broader curricular units, such as fundamental nursing or medical–surgical nursing. Several international authors have identified the positive contributions of specific educational courses/modules on nursing students’ knowledge and attitudes towards HAI prevention and control [10,11,23], especially after the COVID-19 pandemic outbreak [8,24].

Conversely, in this study, nursing students’ perceptions of the HAI prevention and control culture at the healthcare institution where their clinical placements take place influences their motivation to comply with current measures (β = 0.24). Portuguese nursing students often conduct their clinical placement in complex organizational environments. In such environments, institutional resources and tutors’ time are often stretched, leading to the poor execution, or even omission, of crucial HAI prevention and control measures [25,26]. Our findings show that nursing students value receiving guidance and correction by nursing tutors when they breach HAI prevention and control protocols, highlighting the importance of having nursing tutors as role models in this field [26]. These findings compare favorably with international studies, for example, Bouchoucha and colleagues [27] identified the importance of nursing tutors’ leadership and contextual cues as the most important factors influencing nursing students’ self-reported adherence to best practices in this field. Choi and Kim [28] identified that students who conducted their clinical placements in healthcare institutions with an HAI prevention and control structure and dedicated team were more likely to have positive experiences and attitudes towards it.

This study must be interpreted within its limitations. First, we only collected data from convenience samples from two nursing HEIs in Portugal, with a focus on undergraduate nursing students. Thus, careful attention must be paid to the generalization of our findings. Given the implications of this topic to care quality and safety, future studies must consider the inclusion of a more geographically diverse sample (including HEIs from the mainland and islands), as well as including post-graduate nursing students. Second, this is the first instrument on this topic that was translated, culturally adapted, and validated to European Portuguese. The InovSafeCare scale is a self-reported measure composed of 81 items, which can lead to respondent bias due to social desirability and fatigue. Future research must equate other approaches for data collection, testing the psychometric properties of a potentially reduced version of the scale.

Nonetheless, to the best of our knowledge, this is the first study that aimed to evaluate and explore the nursing student’s perceptions regarding their teaching and learning experience in the field of HAI prevention and control in Portugal. We believe this study can inspire discussion on the current pedagogical approaches in this field in nursing curricula, as well as contribute to its revision, and inclusion of specific HAI prevention and control curricular units or courses. 

## 5. Conclusions

The study findings emphasize the need to strengthen the relationship between academic and clinical settings, since nursing students’ knowledge, motivation, and self-efficacy towards HAI prevention and control is influenced during their complementary learning experience in both settings. Nursing teachers and tutors must clearly define common learning goals, strategies, and key experiences in this field, contributing to the development of a learning environment that reinforces among students the fundamental role that nurses have in the prevention and control of HAI. 

## Figures and Tables

**Table 1:** The InovSafeCare scale’s dimensions.

Dimension	Definition
1. Higher Education Institution HAI Theoretical Content (HETheory)	Level of agreement in which specific HAI-related topics are addressed in the Institution.
2. Higher Education Institution Approach to teaching HAI (HEApproach)	Level of agreement in which multiple approaches to teaching about HAI-related topics are used in the Institution.
3. Higher Education Institution HAI practice-related approach/resources (HEPractice)	Level of agreement in which how practice-based approaches are used to address HAI-related topics in the Institution.
4. Health Institution HAI prevention practices (HIPractice)	Degree to which HAI control and prevention safe practices are present in the health center where the student is doing its clinical placement.
5. Health Institution HAI prevention culture (HICulture)	Degree to which HAI safe practices are promoted as values within the health centre where the student realizes its clinical placement.
6. Personal HAI Attitudes (PAttitude)	Degree of agreement in personal attitudes towards HAI prevention and control practices.
7. Personal objection to HAI prevention and control measures (Pobject)	Degree to which the person feels discomfort or objection towards specificities of HAI control and prevention practices.
8. Self-Efficacy on HAI prevention and control (SelfEff)	Degree of confidence the student perceives in its ability to execute HAI safe practices, even under unexpected or stressful conditions.
9. Career Motivation (Cmotivation)	Degree of agreement in how the student feels with their current professional path as a nurse.
10. Fatigue (Fatigue)	Degree to which the student has been experiencing events or phenomena related to exhaustion or fatigue.
11. Distraction (Distract)	Degree to which certain factors may cause distraction or loss of focus in tasks during clinical placements.
12. Intrinsic motives to comply with HAI control and prevention measures (Imotive)	Degree to which the person complies with HAI prevention and control practices because of internal motives.
13. Extrinsic motives to comply with HAI control and prevention measures (Emotive)	Degree to which the person complies with HAI prevention and control practices because of external motives.
14. Self-reported HAI practices (SRPract)	Measures the degree of agreement to which the student reports an HAI-related breach during their clinical placements.

HAI: Healthcare-associated infections.

**Table 2:** Student’s perception about good practice in HAI from Portugal and International sample (including Poland Spain, and Finland).

	International				Portugal			
	Min	Max	Mean	Stand. Dev.	Min	Max	Mean	Stand. Dev.
QI-p1—Chain of Infection	1	5	3.84	1.15	1	5	4.35	0.76
QI-p2—Individual Assessment of risk	1	5	3.81	1.00	1	5	3.95	0.91
QI-p3—Hand Hygiene	1	5	4.82	0.52	1	5	4.87	0.41
QI-p4—Respiratory tract infection	1	5	4.08	0.91	1	5	4.35	0.72
QI-p5—Use of Personal Protective Equipment	1	5	4.29	0.83	2	5	4.51	0.68
QI-p6—Decontamination medical devices	1	5	3.84	1.05	1	5	3.82	0.95
QI-p7—Decontamination of environmental surfaces	1	5	3.72	1.03	1	5	3.75	0.91
QI-p8—Protective Clothing	1	5	3.95	0.97	1	5	3.98	0.91
QI-p9—Clinical waste management	1	5	4.20	0.91	1	5	4.39	0.75
QI-p10—Preparation and administration of intravenous medications	1	5	4.47	0.82	1	5	4.59	0.66
QI-p11—Exposure to microbial agents	1	5	4.05	0.86	1	5	4.19	0.73
QI-p12—Additional Precautions	1	5	3.63	0.98	1	5	3.73	0.89
QII-p1—Mandatory Courses	1	5	3.62	1.13	1	5	3.65	1.12
QII-p2—Theoretical Classes	1	5	4.17	0.88	2	5	4.46	0.68
QII-p3—Workshops or classes	1	5	3.42	1.16	1	5	3.23	1.11
QII-p4—Visual resources and other technology	1	5	3.69	1.07	1	5	3.85	1.00
QII-p5—Teachers discuss HAI measures	1	5	4.14	0.85	2	5	4.32	0.74
QIII-p1—Adequate technological resources	1	5	3.73	1.14	1	5	3.77	1.11
QIII-p2—Student to teacher ratio	1	5	3.59	1.18	1	5	3.43	1.15
QIII-p3—Laboratory Classes	1	5	3.03	1.28	1	5	2.71	1.23
QIII-p4—Simulation	1	5	3.56	1.10	1	5	3.74	1.04
QIII-p5—Feedback from teachers	1	5	3.69	1.01	1	5	3.78	0.93
QIV-p1—Hand hygiene supplies	1	5	4.30	0.88	1	5	4.15	0.95
QIV-p2—Bare below the elbows policy	1	5	3.69	1.26	1	5	3.83	1.17
QIV-p3—Link nurses	1	5	3.10	1.12	1	5	2.94	1.09
QIV-p4—HAI-related resources	1	5	4.07	0.90	1	5	4.05	0.90
QIV-p5—Visual reminders in key areas	1	5	4.38	0.76	1	5	4.34	0.78
QIV-p6—Alcohol-based hand sanitizers key areas	1	5	4.39	0.83	1	5	4.24	0.88
QIV-p7—Personal Protective Equipment key areas	1	5	4.25	0.88	1	5	4.18	0.90
QV-p1—Hand hygiene is promoted	1	5	4.38	0.72	1	5	4.27	0.72
QV-p2—Colleagues follow safety protocols	1	5	3.89	0.81	1	5	3.72	0.77
QV-p3—Working personnel follow safety protocols	1	5	3.58	0.91	1	5	3.39	0.89
QV-p4—Guidance from supervisor	1	5	3.98	0.92	1	5	4.07	0.787
QV-p5—Supervisor makes sure I use PPE	1	5	4.11	0.91	1	5	4.18	0.81
QV-p6—Receive feedback if I do not follow HAI measures	1	5	4.04	0.92	1	5	4.16	0.87
QV-p7—Nursing team motivates me to follow HAI measures	1	5	3.93	0.93	1	5	3.99	0.90
QVI-p1—HAI risk in all moments of patient care	1	5	4.36	0.86	1	5	4.61	0.63
QVI-p2—I feel responsible for HAI prevention and control	1	5	4.70	0.59	2	5	4.74	0.53
QVI-p3—I refuse to take care of patients if I cannot comply with HAI measures	1	5	3.77	1.04	1	5	3.75	1.02
QVI-p4—All HAI protocols must be followed	1	5	4.62	0.63	2	5	4.53	0.64
QVI-p5—Safety measures contribute to prevent infection spread	1	5	4.62	0.61	2	5	4.62	0.56
QVI-p6—I value theoretical recommendations more than my own opinion	1	5	4.00	0.90	1	5	3.88	0.92
QVII-p1—Some HAI protocols are unnecessary	1	5	2.02	0.96	1	5	1.95	0.98
QVII-p2—I express my reservations	1	5	2.13	1.06	1	5	1.96	1.02
QVII-p3—Use of so much disposable material is unnecessary	1	5	2.06	1.09	1	5	1.90	1.00
QVII-p4—PPE is uncomfortable	1	5	2.59	1.096	1	5	2.40	1.11
QVII-p5—My supervisor is constantly monitoring my HAI measures	1	5	2.40	1.171	1	5	2.41	1.15
QVIII-p1—Easy to comply with safety protocols	1	5	4.00	0.78	2	5	4.13	0.68
QVIII-p2—I still follow safety measures	1	5	4.55	0.63	1	5	4.55	0.62
QVIII-p3—I comply with HAI measures under unexpected events	1	5	3.97	0.79	2	5	3.87	0.73
QVIII-p4—I comply with HAI measures under stressful events	1	5	3.96	0.85	1	5	3.89	0.81
QIX-p1—Satisfied with my career choice	1	5	4.33	0.87	1	5	4.27	0.85
QIX-p2—My clinical placements keep me motivated	1	5	4.13	0.97	1	5	4.06	0.93
QIX-p3—Very interested in continuing a career in nursing	1	5	4.48	0.80	1	5	4.44	0.80
QIX-p4—Being a nurse fulfills my expectations	1	5	4.31	0.90	1	5	4.30	0.87
QX-p1—Academic overload	1	5	3.81	0.99	1	5	4.25	0.76
QX-p2—Difficult moments in my personal life	1	5	3.62	1.02	1	5	3.96	0.89
QX-p3—Feeling tired during clinical placements	1	5	3.51	1.03	1	5	3.62	0.96
QX-p4—Emotionally tired	1	5	3.50	1.12	1	5	3.94	0.92
QX-p5—Mentally tired	1	5	3.59	1.08	1	5	3.96	0.88
QX-p6—Physically tired	1	5	3.55	1.04	1	5	3.92	0.88
QXI-p1—Unorganized rooms or units	1	5	2.76	0.96	1	5	2.82	0.98
QXI-p2—Too many patients in the same space	1	5	2.82	1.08	1	5	3.00	1.04
QXI-p3—Number of requests from patients and family	1	5	3.03	0.99	1	5	3.10	0.98
QXI-p4—Talking with peers	1	5	2.61	1.07	1	5	2.48	0.96
QXI-p5—Caring for patients with multiple conditions	1	5	2.86	1.02	1	5	2.81	0.95
QXI-p6—Emotionally demanding cases	1	5	3.03	1.01	1	5	3.17	1.01
QXI-p7—Having to perform multiples clinical tasks	1	5	3.11	1.00	1	5	3.15	0.98
QXII-p1—My main drive is patient health	1	5	4.55	0.62	2	5	4.54	0.60
QXII-p2—I understand its implications	1	5	4.59	0.58	2	5	4.63	0.54
QXII-p3—I aim for quality care	1	5	4.76	0.50	2	5	4.76	0.49
QXII-p4—I am concerned about my patients’ safety	1	5	4.78	0.50	2	5	4.81	0.44
QXII-p5 I am concerned about my own safety	1	5	4.77	0.57	2	5	4.80	0.47
QXII-p6—I want to implement prevention measures	1	5	4.56	0.66	2	5	4.60	0.61
QXII-p7—I am driven by professional ethics	1	5	4.23	0.88	1	5	4.03	0.96
QXII-p8—I will get better grades	1	5	3.52	1.18	1	5	3.35	1.21
QXII-p9—Other team members would think better of me	1	5	3.60	1.17	1	5	3.30	1.20
QXII-p10—Other team members also follow them	1	5	3.66	1.05	1	5	3.37	1.12
QXII-p11—I will meet the expectations of my supervisor	1	5	3.92	1.09	1	5	3.66	1.13
QXIII-p1—Forgotten to follow safety protocols	1	5	2.26	0.98	1	5	2.24	0.95
QXIII-p2—Worn accessories below the elbows	1	5	1.64	1.10	1	5	1.33	0.82
QXIII-p3—Not used gloves	1	5	1.74	1.02	1	5	1.69	0.93
QXIII-p4—Not washed and/or disinfected my hands	1	5	1.65	0.90	1	5	1.72	0.89
QXIII-p5—Not face masks	1	5	1.63	0.93	1	5	1.66	0.89
QXIII-p6—Assisted a patient without changing PPE	1	5	1.56	0.92	1	5	1.41	0.81
QXIV-p1—HAI-dedicated course	1	5	2.74	1.37	1	5	2.46	1.40
QXIV-p2—General courses	1	5	3.67	1.01	1	5	3.77	0.97
QXIV-p3—From my classmates	1	5	3.02	1.08	1	5	3.22	1.03
QXIV-p4—Simulation	1	5	3.37	1.19	1	5	3.32	1.20
QXIV-p5—Ward nurses during clinical placement	1	5	4.02	0.87	1	5	4.03	0.81
QXIV-p6—School supervisors during clinical placement	1	5	3.91	0.97	1	5	3.97	0.90
QXIV-p7—Other professionals during clinical placement	1	5	3.32	1.23	1	5	3.26	1.20
QXIV-p8—Specialized literature	1	5	3.39	1.19	1	5	3.52	1.11
S1_HETheory	1.75	5.00	4.06	0.56	2.00	5.00	4.21	0.48
S2_HEApproach	1.00	5.00	3.81	0.73	1.60	5.00	3.90	0.66
S3_HEPractice	1.00	5.00	3.52	0.84	1.00	5.00	3.49	0.78
S4_HIPractice	1.57	5.00	4.03	0.59	1.57	5.00	3.96	0.61
S5_HICulture	1.00	5.00	3.99	0.64	1.71	5.00	3.97	0.59
S6_Pattitude	1.3	5.0	4.34	0.46	2.0	5.0	4.36	0.44
S7_Pobject	1.00	5.00	2.24	0.75	1.00	5.00	2.12	0.77
S8_SelfEff	1.5	5.0	4.12	0.56	2.0	5.0	4.11	0.52
S9_Cmotivation	1.00	5.00	4.32	0.76	1.00	5.00	4.27	0.74
S10_Fatigue	1.00	5.00	3.60	0.85	1.83	5.00	3.94	0.68
S11_Distract	1.00	5.00	2.89	0.70	1.00	5.00	2.94	0.69
S12_Motive	1.36	5.00	4.27	0.48	2.00	5.00	4.17	0.48
S12A_Imotive	1.00	5.00	4.67	0.42	2.00	5.00	4.69	0.39
S12B_Emotive	1.00	5.00	3.79	0.85	1.00	5.00	3.54	0.90
S13_SRPrac	1.00	5.00	1.74	0.68	1.00	5.00	1.68	0.63
S14_Source	1.00	5.00	3.43	0.66	1.00	5.00	3.44	0.66

Min: minimum value; Max: maximum value; Stand. Dev.: standard deviation.

**Table 3:** Reliability of the InovSafeCare instrument in Portuguese and international sample.

Dimensions	Alpha Cronbach Portuguese Sample	Alpha Cronbach International Sample
1. HETheory	0.85	0.84
2. HEApproach	0.74	0.74
3. HEPractice	0.76	0.76
4. HIPractice	0.76	0.73
5. HICulture	0.84	0.85
6. Pattitude	0.65	0.62
7. Pobject	0.78	0.74
8. SelfEff	0.72	0.72
9. Cmotivation	0.88	0.88
10. Fatigue	0.86	0.89
11. Distract	0.83	0.82
12. Imotive	0.78	0.79
13. SRPract	0.81	0.78
14. Source	0.74	0.73

## Data Availability

The datasets presented in this article are not readily available because the dataset remains for exclusive use by the authors due to participant privacy and informed consent. Requests to access the datasets should be directed to the author of correspondence.
